# Web-based personalised information and support for patients with a neuroendocrine tumour: randomised controlled trial

**DOI:** 10.1186/s13023-019-1035-3

**Published:** 2019-02-28

**Authors:** L. D. de Hosson, G. Bouma, J. Stelwagen, H. van Essen, G. H. de Bock, D. J. A. de Groot, E. G. E. de Vries, A. M. E. Walenkamp

**Affiliations:** 1 Department of Medical Oncology, University of Groningen, University Medical Centre Groningen, DA11, PO Box 30.001, 9700 RB Groningen, The Netherlands; 2 Department of Epidemiology, University of Groningen, University Medical Centre Groningen, Groningen, The Netherlands

**Keywords:** Neuroendocrine tumour, Information, Quality of life, Internet, Web-based system

## Abstract

**Background:**

Patients with a neuroendocrine tumour (NET) frequently have physical and psychosocial complaints. Aim of this study is to determine whether a web-based, personalised information and support system (WINS) reduces distress and/or improves patients’ perception of and satisfaction with information received.

**Methods:**

Patients with NET, stratified for those newly diagnosed (< 6 months, *n* = 28) and with a longer history of disease (*n* = 74), were randomised between standard care (*n* = 49) and intervention, consisting of access to WINS (*n* = 53). Primary outcome was change of distress and satisfaction with perceived information measured with the distress thermometer and problem list and the QoL questionnaire (QLQ)-INFO25. The intervention group also completed a questionnaire based on the technical acceptance model (TAM).

**Results:**

We observed no difference in distress slope and slope of median global score on perceived information and satisfaction between the intervention and control group. Interestingly, 55% of patients wished to receive more information at baseline.

**Conclusions:**

In a population of NET patients, access to WINS did not improve indicators for distress, perception of information and satisfaction with information received, more than standard care only. Despite the need for more information, the WINS does not have added value to the information and care provided by health care professionals.

**Clinical trial registration:**

ClinicalTrials.gov (NCT02472678). Registered 6th Jan 2015. Retrospectively registered 1st May 2017.

**Electronic supplementary material:**

The online version of this article (10.1186/s13023-019-1035-3) contains supplementary material, which is available to authorized users.

## Background and aims

Neuroendocrine tumours (NETs) are rare tumours with an incidence of 3.5/100,000 per year in the last decade [1, 2]. Patients with NET may experience various symptoms from the tumour mass, the output of hormones secreted by the tumour, treatment and accompanying side effects [3]. Patients are frequently metastasized at the time of diagnosis. Patients with metastasized NETs have a relatively long median survival: 100 months for NETs of the small intestine and 60 months for pancreatic NETs [4]. Patients with NET have a lower quality of life (QoL) compared with the general population [5]. For patients with NET it is difficult to find meaningful and understandable information about their diagnosis. Anxiety, higher depression, and stress negatively influenced QoL in patients with NET. Self-efficacy, more social support and optimism are associated with better QoL [6]. Previous research in cancer patients and cancer survivors has consistently shown that high satisfaction with the information received and satisfied information needs were related to better quality of life and lower emotional distress for anxiety and depression [7, 8]. Furthermore internet-based support programs are effective in improving psychosocial and physical symptoms in cancer patients [9]. In addition, in an observational study of NET patients using qualitative interviews, 7 out of 18 patients found the internet to be a useful source of general information [10].

We previously developed a web-based personalised information and support system (WINS), for patients with NETs with the aim of reducing distress and/or improving patients’ perception of and satisfaction with information received.

A pilot study demonstrated the feasibility of using WINS in patients with NET [11]. Based on these results and on patients’ recommendations, we developed the current version of WINS, which was used in the present study. The aim of this randomised trial is to determine whether WINS reduces distress and/or improves NETs patients’ perception of and satisfaction with information received.

## Results

### Patients

**Fig. 1:**
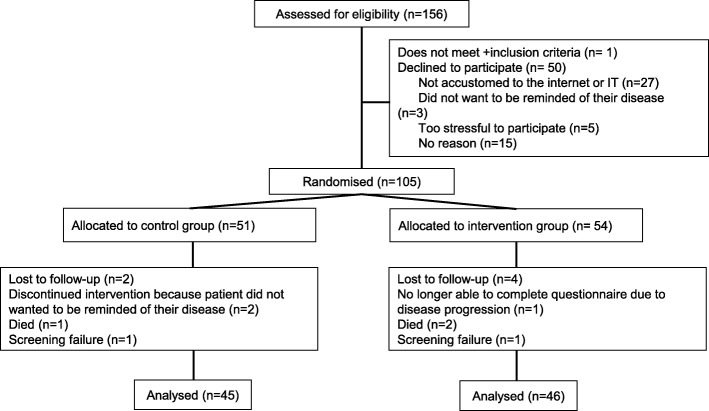
Consort diagram

Between May 2015 and October 2016, we included 105 patients in the study (Fig. 1). The trial was ended when 91 patients completed the study. Of the 91 patients who completed the study, 46 (12 newly diagnosed) patients were randomised to the intervention group. Baseline characteristics of included patients are shown in Table 1. At baseline, 78 patients had previously visited the internet for information on NET before randomisation. At baseline, 50 patients stated that they wanted to receive more information compared to 31 at end of study. At baseline, 4 patients answered that they would prefer to receive less information. At the end of study no patients gave this answer.

**Table 1:** Baseline characteristics

	All patients *n* = 91	Control group *n* = 45	Intervention group *n* = 46
Newly diagnosed patients *n* (%)	25 (27)	13 (29)	12 (26)
Sex (female) *n* (%)	42 (46)	23 (51)	19 (41)
Age mean in years (sd)	62 (8)	63 (7)	62 (10)
Location of primary tumor: *n* (%)			
Pancreas	21 (23)	9 (20)	12 (26)
Intestine	53 (58)	26 (58)	27 (59)
Stomach/duodenum	3 (3)	2 (4)	1 (2)
Colorectal	2 (3)	2 (4)	1 (2)
Bronchopulmonal	1 (1)	1 (2)	0 (0)
Appendix	1 (1)	1 (2)	0 (0)
Unknown/other	9 (10)	4 (9)	5 (11)
Duration of disease in months: mean (SD)	41 (56)	33 (38)	50 (66)
Disease grade: *n* (%)			
1	57 (63)	27 (60)	30 (65)
2	21 (23)	12 (27)	9 (20)
Unknown	13 (14)	6 (13)	7 (15)
Marital state (Married) *n* (%)	75 (82)	34 (76)	41 (89)
Education (Polytechnic or university) *n* (%)	33 (37)	16 (36)	17 (38)
Internet use *n* (%)			
Daily	86 (96)	40 (91)	46 (100)
For information about the disease	78 (87)	37 (82)	41 (89)
Treatment during study: *n* (%)			
Surgery	7 (8)	2 (4)	5 (11)
PRRT	2 (2)	1 (2)	1 (2)
SSA	64 (70)	32 (71)	32 (70)
Systemic treatment other than SSA	17 (19)	9 (20)	8 (17)
Treatment before study: *n* (%)			
Surgery	52 (57)	26 (58)	26 (57)
PRRT	2 (2)	0 (0)	2 (4)
Radiotherapy	3 (3)	0 (0)	3 (6)
SSA	65 (71)	32 (71)	33 (72)
Systemic treatment other than SSA	23 (25)	12 (26)	11 (24)
Other (RFA)	1 (1)	0 (0)	1 (2)

*n* = number, *PRRT* = peptide receptor radionucleide therapy, *RFA* = radiofrequency ablation, *SSA* = somatostatin analogue

### Primary outcomes

The median distress level in both groups was 3 before and after study (Tables 2, 3). A significant difference in distress was found for only the domain ‘social problems’; at end of study 12 patients in the control group of 45, reported social problems compared to 7 patients at baseline. Four patients at end of study in the intervention group of 46 patients, compared to 10 patients at baseline were found to have social problems (*p* < 0.01). The median global score for patients’ perception of and satisfaction with information received, did not improve in the intervention group relative to the control group (Tables 2, 4). Interestingly, 53 and 57% of patients in the control and intervention group wish to receive more information, respectively. After the intervention less patients in the control group (31%) wished to receive more information versus the patients in the intervention group (38%).

**Table 2:** Primary outcome, distress and global score of perceived information and satisfaction (EORTC QLQ-INFO25)

	Control group (*n* = 45)		Intervention group (*n* = 46)		
Outcome	Pre Median (range)	Post Median (range)	Pre Median (range)	Post Median (range)	p
Distress level (0–10)	3 (1–5)	3 (1–5)	3 (2–5)	3 (1–5)	NS
Global Score EORTC QLQ-INFO25 (0–100)	49 (37–55)	51 (42–59)	45 (33–56)	38 (47–57)	NS

Higher scores mean more distress and more/better information and satisfaction

*NS* = no significant difference, *Pre Median* = median score at baseline, *Post median* = median score a 12 weeks, *range* = interquartile range

**Table 3:** Distress (Distress thermometer and Problem List)

	Control group (*n* = 45)		Intervention group (*n* = 46)		
Outcome	Pre Median (range)	Post Median (range)	Pre Median (range)	Post Median (range)	p
Practical problems	0 (0–4)	0 (0–4)	0 (0–4)	0 (0–5)	NS
Social problems	0 (0–0)	0 (0–4)	0 (0–0)	0 (0–0)	0.002
Emotional problems	3.5 (0–13)	6 (0–12)	7 (1–16)	7 (1–16)	NS
Spiritual problems	0 (0–0)	0 (0–0)	0 (0–0)	0 (0–0)	NS
Physical problems	8 (4–18)	10 (6–19)	13 (7–21)	12 (5–20)	NS
Global score	14 (7–32)	17 (6–44)	21 (12–41)	21 (7–44)	NS

Higher scores mean more distress (from problems). The *p*-value shows the differences between the pre-post changes of the control group versus the intervention group

*NS* = no significant difference, *Pre Median*: median score at baseline, *Post median*; median score a 12 weeks, range; interquartile range

**Table 4:** Perceived information and satisfaction (EORTC QLQ-INFO25)

	Control group (*n* = 45)		Intervention group (*n* = 46)		
Outcome	Pre Median (range)	Post Median (range)	Pre Median (range)	Post Median (range)	p
Information about					
Disease	50 (33–58)	50 (42–65)	50 (33–60)	50 (33–67)	NS
Medical tests	67 (44–67)	67 (44–67)	67 (56–67)	67 (28–56)	NS
Treatments	44 (25–56)	39 (33–56)	44 (28–56)	8 (0–27)	NS
Other services	17 (0–25)	17 (8–33)	17 (6–23)	8 (0–27)	NS
Different location of care facilities	0 (0–33)	33 (0–33)	0 (0–33)	0 (0–33)	NS
How to help yourself	33 (0–33)	33 (0–33)	17 (0–33)	33 (0–33)	NS
Satisfaction with information	67 (33–67)	67 (33–67)	67 (33–67)	67 (33–67)	NS
Helpfulness of information	67 (50–67)	67 (67–67)	67 (67–67)	67 (33–67)	NS
Percentage of patients					
Received written information	91	91	78	80	
Received cd/video	2	2	2	0	
Wish to receive more info	53	31	57	38	
Wish to receive less info	2	0	2	0	

Higher scores indicate more/better information and satisfaction. The p-value shows the differences between the pre-post changes of the control group versus the intervention group

*cd* = compact disk, *NS* = no significant difference, *Pre Median* = median score at baseline, *Post median* = median score a 12 weeks, *range* = interquartile range

Most patients agreed with the statements mentioned in the additional questionnaire (Table 5). During the study, the median number of visits to the website was 3 (range 2–4), and only 3 patients used the opportunity to ask questions and consult with the researcher for clinical purposes. Other questions were about technical or logistical aspects.

**Table 5:** Questionnaire based on patients’ opinion and use of the website (based on constructs of the Technology Acceptance Model)

	Intervention group (*n* = 46)
Outcome	Median (range)
The website is useful to me	4 (4–4)
The information at the website is interesting to me	4 (4–4)
I find this a site that adds value	4 (3–5)
I have a positive attitude towards the website	4 (4–5)
I would recommend the site to peers	4 (3–5)
How often do you visit the website	3 (2–4)

Higher scores indicate more agreement with the statement (except for number of visits)

Median: median score (at 12 weeks), range; interquartile range

### Newly diagnosed patients

The planned subgroup analysis for newly diagnosed patients did not detect any difference in this subgroup regarding the global score for distress and the problem domains (Additional file 1 and Additional file 2: Tables S1 and S2).

In this subgroup, perceived information and satisfaction did not differ between the control group and intervention group. We found a difference for only one item, ‘information about the disease in the QLQ-INFO25’; after 12 weeks this score decreased in the intervention group and increased in the control group (*p* = 0.046). Most patients agreed with the statements on the self-constructed questionnaire (Additional file 1 and Additional file 2: Table S5).

## Discussion

In this randomised trial we found that WINS did not reduce distress nor improved NETs patients’ perception of and satisfaction with information received. We found the same results in the pre-planned subgroup analyses with newly diagnosed patients.

Developing a web-based system (WBS) providing patient detailed information corresponding to their individual needs and wishes is difficult [12]. The contact and communication with health care professionals remains a crucial source of information and support for patients with NET. Other web-based studies have shown that web-based technology does not replace patient-provider communication [13]. In one such study, in which 103 cancer patients received questionnaires about the WBS, the highest rated component was receiving an answer from a nurse. In another trial in 766 patients receiving chemotherapy for solid cancers, patients were randomised to either standard care or to standard care plus a web-based self-reporting system. Physicians received symptom printouts at visits and nurses received e-mail alerts when participants of the intervention group reported severe or worsening symptoms. In this trial, QoL improved (34% vs. 18%; *p* < 0.001) and OS was higher (31 vs. 26 months; *p* = 0.03) in the intervention group compared to the group receiving standard care only [14]. Another example, that web-based technology does not replace patient-provider communication, was presented in a randomised controlled study of a website providing additional information and symptom self-management support in 325 breast and prostate cancer patients. Distress was used as the primary endpoint [15]. Compared to the control group, which was given uniform resource locators (URLs) of publically available cancer information websites, an improvement for patients in the intervention group was found only on the subscale ‘global distress index’.

A potential explanation for these findings could be that NET patients obtain sufficient information by usual care that all patients received. In our trial all patients had easy access to specialists and nurses during standard care, which might have satisfied their need for information. Furthermore, patients were able to get information from the NET patient association [16]. We did not use a control group receiving no information which can be regarded as a limitation of our study.

At baseline half of the patients in both groups indicated that they wanted more information about the disease. During study period, the need for more information decreased in both groups. A decline in the need for information during the course of the disease was observed in other studies analysing patients with other types of cancer [17, 18]. Furthermore, patients only visited the WBS sporadically. Patients were not requested to visit the website more frequent, which also could be seen as a limitation. Sporadically visiting a WBS was also reported in other trials using a WBS [15, 19]. In a qualitative interview study, one of the three main reasons reported for not using a WBS was that patients had sufficient access to information elsewhere [20]. In two other studies, in patients with cancer and brain tumours, patients reported that they avoided using the WBS because they did not want to be reminded of their suffering or, because they had a preference for other kinds of communication [20, 21].

Another limitation is that we could only analyze patients with outcome data on the second assessment, as the primary endpoint was the pre-post change of distress and satisfaction with perceived information. For that, the 14 (13%) of the patients who withdrew early, or who did not complete the end-of-study questionnaires were excluded from the analysis.

## Conclusion

In NET patients, WINS did not improve distress scores and patients’ perception of or satisfaction with information received, compared to patients receiving standard care only. Despite the need for more information, the WINS does not have added value to the information and care provided by health care professionals.

## Methods

### Participants

Eligible participants were adult patients treated at the University Medical Centre Groningen Department of Medical Oncology for NET grade 1 or 2 (World Health Organization 2010 classification) with the primary tumour at any site of origin, and who were proficient in Dutch (both reading and writing). Patients with a life expectancy of less than 3 months as evaluated by their doctor were excluded. The study was approved by the medical ethical committee of the UMCG and registered in ClinicalTrials.gov (NCT02472678). All patients gave written informed consent.

### Randomisation process and study procedure

For a detailed description of study procedures, see Additional file 1 and Fig. 1. The included patients were stratified randomised for those diagnosed within 6 months and those with disease duration ≥6 months. Patients were randomised 1:1 to the control group, receiving standard care, or the intervention group, which received standard care with additional access to WINS. At baseline patients’ socio-demographic and disease characteristics, internet use and health care use were collected. Patients in both groups received a questionnaire about their perception of and satisfaction with the received information and their QoL. The control group was also given a questionnaire about distress and problems. After returning the questionnaires, patients in the intervention group received log-in information for website access. At the first website visit, the patients were asked to complete a questionnaire about distress and problems at baseline, before they were given access to the other items on the website. At 12 weeks follow-up, all patients were asked to complete the questionnaires again, with an additional questionnaire to asses empowerment. Patients in the intervention group were asked to complete the questionnaire about distress and problems at the WINS instead of completing it with pen and paper. Furthermore, they were requested to complete an additional questionnaire about their use of and opinion about WINS.

### Standard care and study intervention

All patients received standard care. At their first visit at the Department of Medical Oncology patients are informed verbally about the disease and they meet the oncology nurse to become acquainted. During follow-up visits, the medical oncologist evaluates the general well-being, discusses possible treatment options and treatment side-effects (if applicable) and answers questions of the patient. Between follow-up visits all patients could consult an oncology nurse 24 h a day, 7 days a week by telephone. Furthermore, patients were able to get information from the NET patient association [16]. If physical and/or psychosocial problems require more in-depth discussion, investigation or treatment, patients can receive a consultation with the oncology nurse, oncologist or other health care professionals.

In the intervention group, the questionnaire about distress and problems served as a self-screening tool for physical and psychosocial problems. By using WINS, patients could obtain personalised information about reported problems. Self-screening was performed by the online version of the Dutch Distress Thermometer (DT) and Problem List (PL). Immediately after completion of the DT and PL. Patients received online information on the physical and psychosocial problems they reported on the digital PL. This information comprises: a) description and background information of their reported problem b) advice on how to cope with the problem (self-help) c) what health care professional could be consulted when self-help insufficiently alleviates problems. On the WINS, patients could also find general information about the disease, read about the experiences of other NET patients and find links to other relevant websites. Any time, patients could send an e-mail with a question or request a telephone consultation with the investigators (physicians experienced in treating NET patients) in case of questions, problems or request further referral’.

### Outcome measurements

Illness-related patient characteristics were extracted from the medical records at baseline. All other measurements were performed by self-report questionnaires. Selection of endpoints was based on the results of the pilot study [11]. According to our pre planned protocol our primary endpoint was the combined endpoint of distress the change in distress level combined with change in global score of perception of and satisfaction with information received. Distress was measured using the validated Dutch Distress Thermometer (DT) and Problem list (PL) [22]. This questionnaire consisted of one single item that asks patients to indicate the amount of overall distress experienced during the past week and a PL with several items divided into five domains. Higher scores indicate more distress or more problems. The EORTC QLQ-INFO25 in Dutch was used to evaluate patients’ perception of and satisfaction with information received [23]. Higher scores indicate better perceived information provision. The measures of the validated QLQ-INFO25 are categorized and scored according to the EORTC guidelines. For a more detailed descripition of the questionnaires, see our pilot study. An additional questionnaire, only given to the intervention group at end of study was based on the constructs of ‘perceived usefulness’ and ‘attitude and usage’ from the revised Technology Acceptance Model and on a self-constructed question on a 5-point likert scale; if patients recommend WINS to other NET patients [24].

### Sample size calculation

Sample size calculation was based on the results of the pilot study, in which only newly diagnosed patients were included. To detect a significant difference in the change of the distress thermometer between the control and intervention group, using an independent t-test with an effect size of 0.6, we calculated that 90 patients had to be included (Additional file 1). Taking into account a dropout of 15%, we included 105 patients in this study,

### Statistical analysis

For descriptive statistics, mean and standard deviation (sd) for normal distribution and median and interquartile range for other distributions and frequencies were calculated for all measures. The scores of the EORTC questionnaires were calculated according to the EORTC guidelines [23, 25, 26]. An independent t-test or Mann Whitney U test was performed, depending on the kind of distribution, to detect differences between the pre-post changes of standard care complemented with the WINS versus standard care alone. Given the negative findings of the outcome we did not correct for multiple comparison issues. Subgroup analysis was performed in newly diagnosed patients. Differences were considered significant at *p* < 0.05. Analyses were performed with the software package SPSS, version 23 for Windows (SPSS, Inc., Chicago, IL, USA). Patients who were lost to follow up were excluded from analysis.

## Additional files

Additional file1: Supportive Information. (DOCX 24 kb)

Additional file 2: **Table S1.** Distress in newly diagnosed patients (Distress thermometer and Problem List). **Table S2.** Perceived information and satisfaction with information in newly diagnosed patients (EORTC QLQ-INFO25). **Table S3A.** Quality of life in newly diagnosed patients (EORTC QLQ-C30). **Table S3B.** Quality of life in newly diagnosed patients (EORTC QLQ-GINET21). **Table S4.** Empowerment in newly diagnosed patients (CEO). **Table S5.** Questionnaire on patients’ opinions and use of the website in newly diagnosed patients (based on constructs of the Technology Acceptance Model). **Table S6A.** Quality of life (EORTC QLQ-C30). **Table S6B.** Quality of life (EORTC QLQ-GINET21). **Table S7.** Empowerment (CEO). (DOCX 50 kb)

## Abbreviations

CEO: Constructs empoering outcomes
DT: Distress
EORTC: Eoropean organization for research and treatment of cancer
N: Number
NET: Neuroendocrine tumor
PL: Problem list
QLQ: Quality of life questionnaire
QoL: Quality of life
RCT: Randomised controlled trial
SD: Standard deviation
SI: Supportive information
TAM: Technical acceptance model
WBS: Web-based system
WINS: Web-based personalised information and support system
