# Supplementary material for: Web-based personalised information and support for patients with a neuroendocrine tumour: randomised controlled trial
Source: Orphanet J Rare Dis. 2019 Feb 28;14:60. doi: 10.1186/s13023-019-1035-3 (PMC6394034; doi:10.1186/s13023-019-1035-3)
Supplement: Supplementary file 1 — Supportive Information. (DOCX 24 kb) [file 13023_2019_1035_MOESM1_ESM.docx]

**Supportive Information**

**Randomisation process and study procedure**The included patients were stratified randomised for those diagnosed within 6 months and those with disease duration ≥6 months. We expected that newly diagnosed patients would have other information needs which could lead to bias if not stratified. Patients were randomised 1:1 to the control group, receiving standard care, or the intervention group, which received standard care with additional access to WINS. Randomisation was performed by the central data manager of the Department of Medical Oncology (UMCG) who was not involved in other aspects of the study. A computer-generated randomisation list was used for allocation to the control or intervention arm. The allocation sequence was concealed from the investigator. The investigator was informed by e-mail to which arm the patient was assigned. The investigator informed the patient by phone about the randomisation outcome and further process. At baseline patients’ socio-demographic and disease characteristics, internet use and health care use were collected. Patients in both groups received a questionnaire about their perception of and satisfaction with the received information and their QoL. The control group was also given a questionnaire about distress and problems. After returning the questionnaires, patients in the intervention group received log-in information for website access. At the first website visit, the patients were asked to complete a questionnaire (within 1 week) about distress and problems at baseline, before they were given access to the other items on the website. At 12 weeks follow-up, all patients were asked to complete the questionnaires again, with an additional questionnaire to asses empowerment. Patients in the intervention group were asked to complete the questionnaire about distress and problems at the WINS instead of completing it with pen and paper. Furthermore they were requested to complete an additional questionnaire about their use of and opinion about WINS.

**Outcome measurements**

QoL was measured by the cancer-specific Dutch EORTC QLQ-C30 (version 3.0) and the NET-specific EORTC QLQ-GINET21 (1,2). Higher scores indicate higher QoL at the functional and global health/QoL scale and higher symptom burden for the symptom scales of the QLQs(1). The measures of the validated QLQ-INFO25, QLQ-C30 and QLQ-GINET-21 are categorized and scored according to the EORTC guidelines. The significance of change in QoL is analysed for the QLQ-C30 and for patients who indicated "a little" change either for better or for worse, the mean change in scores was about 5 to 10; for "moderate" change, about 10 to 20; and for "very much" change, greater than 20 (3). Empowerment was measured with specific domains of the ‘Constructs Empowering Outcomes’ (CEO) questionnaire(4). Higher scores indicate greater empowerment(4). This questionnaire has a retrospective nature. Furthermore, this questionnaire has not been extensively validated. Consequently, our findings should be interpreted with caution(4-6).

**Sample size calculation**

Sample size calculation was based on the results of the pilot study, in which only newly diagnosed patients were included. The score of distress level had a Cohen’s d effect size of 0.75 in favour of the intervention group and an effect size of 1.0 was found in favour of the control group for the global score of the EORTC QLQ-INFO25. Since we expected using a WINS for newly diagnosed patients and for patients diagnosed more than 6 months before inclusion, might have a smaller effect, than for only newly diagnosed patients, we adapted the effect size to 0.6 for the distress thermometer and to 0.8 fot the EORTC QLQ-INFO 25. To detect a significant difference in the change of the distress thermometer between the control and intervention group, using an independent t-test with an effect size of 0.6, we calculated that 90 patients had to be included.

**Results**

No significant differences were found between the control and intervention groups for each symptom, problem, or level of functioning regarding QoL (Table S6). However power analysis was not performed to detect a difference in empowerment, empowerment was better in the control group, for all constructs, except for ‘optimism and control over future’ for which no difference was found (Table S7). Most patients agreed with the statements mentioned in the additional questionnaire (Table 5). During the study, the median number of visits to the website was 3 (range 2-4), and only 3 patients used the opportunity to ask questions and consult with the researcher for clinical purposes. Other questions were about technical or logistical aspects.

**Newly diagnosed patients**

Furthermore, regarding the secondary endpoints, QoL and empowerment, most items did not show significant differences were found between the control and intervention groups. Most patients agreed with the statements on the self-constructed questionnaire (SI Tables S3-S5).

**Discussion**

In our study, empowerment was better in the control group. In other trials in cancer patients no statistically differences were seen in empowerment between the control group and the intervention group with access to the web-based intervention(7,8)

Due to the indolent natural course of NET, and because NET patients may not have symptoms at study inclusion, it is challenging to demonstrate an improvement in QoL in this population(9). In the RADIANT-4, a large randomised controlled trial (RCT) on the use of everolimus in advanced non-functional, gastrointestinal or lung NET patients, few patients had symptoms at study inclusion. During the study period, global QoL remained stable in both groups, as also seen in our previous study(10).

Despite its well-powered prospective randomised controlled design, our study also has some limitations, in particular the use of the constructs empowering outcome (CEO) questionnaire. Due to the retrospective nature of the CEO questionnaire, no baseline scores could be determined. Furthermore, this questionnaire has not been extensively validated. Consequently, our finding that empowerment at the end of the study was better in the control group should be interpreted with caution(4-6).

References

1. Aaronson NK, Ahmedzai S, Bergman B, et al. The European Organization for Research and Treatment of Cancer QLQ-C30: a quality-of-life instrument for use in international clinical trials in oncology. J Natl Cancer Inst 1993;85:365-76.

2. Yadegarfar G, Friend L, Jones L, et al. Validation of the EORTC QLQ-GINET21 questionnaire for assessing quality of life of patients with gastrointestinal neuroendocrine tumours. Br J Cancer 2013;108:301-10.

3. Osoba D, Rodrigues G, Myles J, Zee B, Pater J. Interpreting the significance of changes in health-related quality-of-life scores. J Clin Oncol 1998;16:139-44.

4. van Uden-Kraan CF, Drossaert CH, Taal E, Shaw BR, Seydel ER, van de Laar MA. Empowering processes and outcomes of participation in online support groups for patients with breast cancer, arthritis, or fibromyalgia. Qual Health Res 2008;18:405-17.

5. Admiraal JM, van der Velden AWG, Geerling JI, et al. Web-based tailored psycho-education for breast cancer patients at the onset of the survivorship phase: a multicenter randomized controlled trial. J Pain Symptom Manage 2017;:Epub ahead of print.

6. van Uden-Kraan CF, Drossaert CH, Taal E, Seydel ER, van de Laar MA. Participation in online patient support groups endorses patients' empowerment. Patient Educ Couns 2009;74:61-9.

7. Admiraal JM, van der Velden AWG, Geerling JI, et al. Web-Based tailored psychoeducation for breast cancer patients at the onset of the survivorship phase: A multicenter randomized controlled trial. J Pain Symptom Manage 2017;54:466-75.

8. van den Berg SW, Gielissen MF, Custers JA, van der Graaf WT, Ottevanger PB, Prins JB. BREATH: Web-based self-management for psychological adjustment after primary breast cancer--results of a multicenter randomized controlled trial. J Clin Oncol 2015;33:2763-71.

9. Dasari A, Shen C, Halperin D, et al. Trends in the Incidence, Prevalence, and Survival Outcomes in Patients With Neuroendocrine Tumors in the United States. JAMA Oncol 2017;3:1335-42.

10. Bouma G, de Hosson LD, van Woerkom CE, et al. Web-based information and support for patients with a newly diagnosed neuroendocrine tumor: a feasibility study. Support Care Cancer 2017;25:2075-83.
