# Supplementary material for: Web-based personalised information and support for patients with a neuroendocrine tumour: randomised controlled trial
Source: Orphanet J Rare Dis. 2019 Feb 28;14:60. doi: 10.1186/s13023-019-1035-3 (PMC6394034; doi:10.1186/s13023-019-1035-3)
Supplement: Supplementary file 2 — Table S1. Distress in newly diagnosed patients (Distress thermometer and Problem List). Table S2. Perceived information and satisfaction with information in newly diagnosed patients (EORTC QLQ-INFO25). Table S3A. Quality of life in newly diagnosed patients (EORTC QLQ-C30). Table S3B. Quality of life in newly diagnosed patients (EORTC QLQ-GINET21). Table S4. Empowerment in newly diagnosed patients (CEO). Table S5. Questionnaire on patients’ opinions and use of the website in newly diagnosed patients (based on constructs of the Technology Acceptance Model). Table S6A. Quality of life (EORTC QLQ-C30). Table S6B. Quality of life (EORTC QLQ-GINET21). Table S7. Empowerment (CEO). (DOCX 50 kb) [file 13023_2019_1035_MOESM2_ESM.docx]

**Table S1. Distress in newly diagnosed patients (Distress thermometer and Problem List)**

|  | Control group (n=13) | | Intervention group (n=12) | | |
| --- | --- | --- | --- | --- | --- |
| Outcome | **Pre**  **Median (range)** | **Post**  **Median (range)** | **Pre**  **Median (range)** | **Post**  **Median (range)** | **p** |
| Distress level (0-10) | 4 (2-6) | 5 (2-7) | 3 (0-3) | 3 (0-3) | NS |
| Practical problems | 0 (0-3) | 0 (0-5) | 0 (0-0) | 0 (0-2) | NS |
| Social problems | 0 (0-0) | 0 (0-10) | 0 (0-0) | 0 (0-0) | NS |
| Emotional problems | 0 (4-12) | 0 (0-16) | 3 (0-15) | 3 (0-8) | NS |
| Spiritual problems | 0 (0-2) | 0 (0-0) | 0 (0-0) | 0 (0-0) | NS |
| Physical problems | 12 (7-18) | 14 (5-24) | 8 (4-14) | 11 (2-15) | NS |
| Global score | 15 (13-33) | 35 (9-57) | 16 (7-40) | 14 (3-27) | NS |

Higher scores indicate more distress (from problems). The p-value shows the differences between the pre-post changes of the control group versus the intervention group. NS= no significant difference, Pre Median: median score at baseline, Post median: median score at 12 weeks, range: interquartile range.

**Table S2. Perceived information and satisfaction with information in newly diagnosed patients (EORTC QLQ-INFO25)**

|  | Control group (n=13) | | | | Intervention group (n=12) | | |
| --- | --- | --- | --- | --- | --- | --- | --- |
| Outcome | **Pre**  **Median (range)** | | | **Post**  **Median (range)** | **Pre**  **Median (range)** | **Post**  **Median (range)** | **p** |
| Information about | | | | | | | |
| Disease | | 42 (38-63) | 58 (46-67) | | 50 (27-73) | 38 (25-67) | 0.046 |
| Medical tests | | 67 (50-67) | 67 (56-67) | | 56 (33-67) | 67 (44-67) | NS |
| Treatments | | 33 (22-53) | 39 (31-61) | | 42 (24-54) | 40 (22-61) | NS |
| Other services | | 4 (17-29) | 25 (4-50) | | 13 (0-25) | 8 (0-37) | NS |
| Different location of care facilities | | 0 (0-33) | 25 (4-50) | | 0 (0-33) | 0 (0-58) | NS |
| How to help yourself | | 33 (0-33) | 33 (0-50) | | 33 (0-33) | 33 (8-58) | NS |
| Satisfaction with information | | 67 (33-67) | 67 (50-67) | | 67 (33-67) | 67 (33-100) | NS |
| Helpfulness of information | | 67 (50-67) | 67 (67-67) | | 67 (67-67) | 67 (33-100) | NS |
|  | | **Percentage of patients** | | | | | |
| Received written information | | 100 | 100 | | 100 | 82 |  |
| Received cd/video | | 0 | 8 | | 0 | 0 |  |
| Wish to receive more info | | 54 | 15 | | 58 | 64 |  |
| Wish to receive less info | | 0 | 0 | | 0 | 0 |  |
| Outcome | | **Pre M (SD)** | **Post M (SD)** | | **Pre M (SD)** | **Post M (SD)** | **p** |
| Global Score | | 52 (41-57) | 55 (52-62) | | 48 (39-56) | 42 (31-63) | NS |

Higher score indicates more/better information and satisfaction. The p-value shows the differences between the pre-post changes of the control group versus the intervention group. cd: compact disk, NS: no significant difference, Pre Median: median score at baseline, Post median” median score a 12 weeks, range: interquartile range.

**Table S3A. Quality of life in newly diagnosed patients (EORTC QLQ-C30)**

|  | Control group (n=13) | | Intervention group (n=12) | | | | |
| --- | --- | --- | --- | --- | --- | --- | --- |
| Outcome | **Pre**  **Median (range)** | **Post**  **Median (range)** | **Pre**  **Median (range)** | | **Post**  **Median (range)** | | **p** |
| Global quality of life | 75 (50-83) | 75 (54-83) | 75 (69-83) | | 75 (67-83) | | NS |
|  | | | | | | | |
| Physical | 80 (53-100) | 80 (53-93) | 93 (87-100) | 87 (82-98) | | NS | |
| Role | 67 (42092) | 67 (67-100) | 83 (67-100) | 83 (53-100) | | NS | |
| Emotional | 83 (75-100) | 83 (63-96) | 79 (67-92) | 79 (67-100) | | NS | |
| Cognitive | 67 (67-100) | 67 (67-92) | 100 (83-100) | 75 (67-100) | | NS | |
| Social | 83 (50-100) | 83 (67-100) | 100 (83-100) | 83 (67-100) | | NS | |
|  | | | | | | | |
| Dyspnea | 0 (0-33) | 0 (0-50) | 0 (0-0) | 0 (0-0) | | NS | |
| Insomnia | 0 (0-33) | 33 (17-33) | 33 (0-33) | 33 (0-33) | | NS | |
| Appetite loss | 0 (0-33) | 0 (0-0) | 0 (0-0) | 0 (0-0) | | NS | |
| Constipation | 0 (0-0) | 0 (0-0) | 0 (0-0) | 0 (0-0) | | NS | |
| Diarrhea | 0 (0-33) | 33 (0-67) | 0 (0-33) | 33 (0-58) | | NS | |
| Financial difficulties | 0 (0-0) | 0 (0-0) | 0 (0-0) | 0 (0-0) | | NS | |
| Fatigue | 44 (6-56) | 33 (22-44) | 17 (3-31) | 22 (11-61) | | NS | |
| Nausea and vomiting | 0 (0-8) | 0 (0-17) | 0 (0-0) | 0 (0-0) | | NS | |
| Pain | 33 (0-75) | 33 (8-42) | 8 (0-29) | 0 (0-33) | | NS | |

Higher scores for quality of life and functioning indicate higher quality of life and level of functioning. Higher scores for symptoms indicate greater severity. The p-value shows the differences between the pre-post changes of the control group versus the intervention group.

NS: no significant difference, Pre Median: median score at baseline, Post median: median score a 12 weeks, range: interquartile range.

**Table S3B. Quality of life in newly diagnosed patients (EORTC QLQ-GINET21)**

|  | Control group (n=13) | | | Intervention group (n=12) | | |
| --- | --- | --- | --- | --- | --- | --- |
| Outcome | | **Pre**  **Median (range)** | **Post**  **Median (range)** | **Pre**  **Median (range)** | **Post**  **Median (range)** | **p** |
| Endocrine symptoms | | 11 (0-33) | 0 (0-19) | 0 (0-19) | 0 (0-8) | NS |
| Gastrointestinal symptoms | | 20 (3-40) | 27 (9 -37) | 17 (13-20) | 17 (13-32) | NS |
| Treatment related symptoms | | 16 (6-28) | 22 (6-33) | 14 (0-22) | 0 (0-22) | NS |
| Problems social functioning | | 33 (28-67) | 33 (17-61) | 33 (25-53) | 33 (22-53) | NS |
| Disease-related worries | | 33 (19-72) | 33 (25-72) | 53 (36-67) | 33 (17-44) | 0.006 |
| Pain muscles/bone | | 33 (0-50) | 33 (8-58) | 0 (0-25) | 0 (0-33) | NS |
| Problems sexual functioning | | 0 (0-58) | 17 (0-75) | 0 (0-33) | 0 (0-42) | NS |
| Problems receiving information | | 0 (0-33) | 0 (0-0) | 0 (0-33) | 0 (0-0) | NS |
| Problems body image | | 0 (0-33) | 0 (0-33) | 0 (0-0) | 0 (0-0) | NS |

Higher scores indicate more or worse symptoms/problems. The p-value shows the differences between the pre-post changes of the control group versus the intervention group. NS: no significant difference, Pre Median: median score at baseline, Post median: median score at 12 weeks, range: interquartile range.

**Table S4. Empowerment in newly diagnosed patients (CEO)**

|  | Control group (n=13) | Intervention group (n=12) |  |
| --- | --- | --- | --- |
| Outcome | **Median (range)** | **Median (range)** |  |
| Feeling informed | 16 (14-18) | 14 (13-16) |  |
| Confidence in relationship physician | 34 (33-42) | 31 (25-39) |  |
| Confidence in treatment | 19 (16-20) | 16 (10-19) |  |
| Acceptance of illness | 17 (15-20) | 13 (9-20) |  |
| Optimism and control over future | 24 (23-28) | 16 (14-26) |  |

Higher scores indicate better empowerment for each outcome. The p-value shows the differences between the pre-post changes of the control group versus the intervention group.

NS: no significant difference, Median: median score (at 12 weeks), range: interquartile range.

**Table S5. Questionnaire on patients’ opinions and use of the website in newly diagnosed patients (based on constructs of the Technology Acceptance Model)**

|  | Intervention group (n=12) |
| --- | --- |
| Outcome | **Median (range)** |
| The website is useful to me | 4 (4-5) |
| The information at the website is interesting to me | 4 (3-4) |
| I find this a site that adds value | 4 (4-5) |
| I have a positive attitude towards the website | 4 (4-5) |
| I would recommend the site to peers | 4 (3-5) |
| How often do you visit the website | 4 (3-5) |

Higher scores indicate more agreement with the statement except for number of visits

Median: median score (at 12 weeks), range: interquartile range.

**Table S6A. Quality of life (EORTC QLQ-C30)**

|  | Control group (n=45) | | Intervention group (n=46) | | | | |
| --- | --- | --- | --- | --- | --- | --- | --- |
| Outcome | **Pre**  **Median (range)** | **Post**  **Median (range)** | **Pre**  **Median (range)** | | **Post**  **Median (range)** | | **p** |
| Global quality of life | 75 (58-83) | 75 (62-83) | 75 (67-83) | | 75 (65-83) | | NS |
|  | | | | | | | |
| Physical | 87 (67-100) | 87 (67-97) | 87 (73-100) | 87 (72-100) | | NS | |
| Role | 83 (50-100) | 83 (67-100) | 83 (63-100) | 83 (67-100) | | NS | |
| Emotional | 92 (75-100) | 83 (67-96) | 83 (67-96) | 83 (67-94) | | NS | |
| Cognitive | 100 (67-100) | 83 (67-100) | 100 (83-100) | 100 (67-100) | | NS | |
| Social | 100 (67-100) | 100 (83-100) | 100 (67-100) | 83 (67-100) | | NS | |
|  | | | | | | | |
| Dyspnea | 0 (0-0) | 0 (0-33) | 0 (0-0) | 0 (0-0) | | NS | |
| Insomnia | 0 (0-33) | 33 (0-33) | 33 (0-33) | 33 (0-33) | | NS | |
| Appetite loss | 0 (0-33) | 0 (0-0) | 0 (0-0) | 0 (0-0) | | NS | |
| Constipation | 0 (0-0) | 0 (0-0) | 0 (0-0) | 0 (0-0) | | NS | |
| Diarrhea | 0 (0-33) | 0 (0-50) | 0 (0-33) | 0 (0-0) | | NS | |
| Financial difficulties | 0 (0-0) | 0 (0-0) | 0 (0-0) | 0 (0-0) | | NS | |
| Fatigue | 33 (17-56) | 22 (11-44) | 22 (11-39) | 28 (11-44) | | NS | |
| Nausea and vomiting | 0 (0-17) | 0 (0-8) | 0 (0-0) | 0 (0-0) | | NS | |
| Pain | 0 (0-33) | 17 (0-33) | 17 (0-33) | 17 (0-33) | | NS | |

Higher scores for quality of life and functioning indicate higher quality of life and level of functioning. Higher scores for symptoms indicate greater severity. The p-value shows the differences between the pre-post changes of the control group versus the intervention group. NS= no significant difference, Pre Median= median score at baseline, Post median=median score a 12 weeks, range= interquartile range.

**CONTINUED**

**Table S6B. Quality of life (EORTC QLQ-GINET21)**

|  | Control group (n=45) | | | Intervention group (n=46) | | |
| --- | --- | --- | --- | --- | --- | --- |
| Outcome | | **Pre**  **Median (range)** | **Post**  **Median (range)** | **Pre**  **Median (range)** | **Post**  **Median (range)** | **p** |
| Endocrine symptoms | | 11 (0-33) | 11 (0-33) | 0 (0-14) | 11 (0-33) | NS |
| Gastrointestinal symptoms | | 20 (7-27) | 20 (12-33) | 20 (13-28) | 20 (7-33) | NS |
| Treatment related symptoms | | 11 (0-22) | 17 (0-28) | 11 (0-22) | 0 (0-22) | NS |
| Problems social functioning | | 33 (17-44) | 22 (11-33) | 33 (22-56) | 22 (11-44) | NS |
| Disease related worries | | 33 (19-53) | 33 (17-50) | 44 (22-67) | 33 (19-44) | NS |
| Pain muscles/bone | | 33 (0-33) | 33 (0-33) | 33 (0-67) | 33 (0-33) | NS |
| Problems sexual functioning | | 0 (0-50) | 0 (0-33) | 17 (0-33) | 0 (0-67) | NS |
| Problems receiving information | | 0 (0-0) | 0 (0-0) | 0 (0-33) | 0 (0-0) | NS |
| Problems body image | | 0 (0-33) | 0 (0-33) | 0 (0-33) | 0 (0-0) | NS |

Higher scores indicate more or worse symptoms/problems. NS= no significant difference, Pre Median: median score at baseline, Post median; median score a 12 weeks, range; interquartile range.

**Table S7. Empowerment (CEO)**

|  | Control group (n=45) | Intervention group (n=46) |  |
| --- | --- | --- | --- |
| Outcome | **Median (range)** | **Median (range)** |  |
| Feeling informed | 16 (14-16) | 13 (12-14) |  |
| Confidence in relationship physician | 37 (34-41) | 32 (26-37) |  |
| Confidence in treatment | 19 (16-20) | 15 (12-18) |  |
| Acceptance of illness | 18 (15-20) | 15 (11-17) |  |
| Optimism and control over future | 24 (22-26) | 23 (16-25) |  |

Higher scores represent better empowerment for each outcome. NS= no significant difference, Median: median score (at 12 weeks), range; interquartile range.
